# Unmet needs and wish for support of family caregivers of primary brain tumor patients

**DOI:** 10.1093/nop/npac099

**Published:** 2023-01-04

**Authors:** Lucy Pointon, Robin Grant, Sharon Peoples, Sara Erridge, Paula Sherwood, Martin Klein, Florien Boele

**Affiliations:** Leeds Institute of Medical Research at St James’s, University of Leeds, Beckett Street, Leeds LS9 7TF, UK; School of Psychology, University of Leeds, University Rd, Woodhouse, Leeds LS2 9JU, UK; Edinburgh Centre for Neuro-Oncology, Western General Hospital Edinburgh Cancer Centre, Edinburgh EH4 2LF, UK; Edinburgh Centre for Neuro-Oncology, Western General Hospital Edinburgh Cancer Centre, Edinburgh EH4 2LF, UK; Edinburgh Centre for Neuro-Oncology, Western General Hospital Edinburgh Cancer Centre, Edinburgh EH4 2LF, UK; University of Pittsburgh, School of Nursing, 336 Victoria Bldg, Pittsburgh, Pennsylvania 15261, USA; Amsterdam UMC Location Vrije Universiteit Amsterdam, Medical Psychology, PO Box 7057, 1007 MB Amsterdam, The Netherlands; Cancer Center Amsterdam, Brain Tumor Center, PO Box 7057, 1007 MB Amsterdam, The Netherlands; Leeds Institute of Medical Research at St James’s, University of Leeds, Beckett Street, Leeds LS9 7TF, UK; Leeds Institute of Health Sciences, University of Leeds, Leeds LS2 9NL, UK

**Keywords:** brain tumor, caregiver needs, neuro-oncology, screening instrument

## Abstract

**Background:**

Most primary brain tumor patients rely on family caregivers for support. Caregiving can be rewarding, but also leads to significant burden from unmet needs. We aimed to: (1) identify and characterize caregivers’ unmet needs; (2) determine associations between unmet needs and wish for support; (3) evaluate acceptability of the Caregiver Needs Screen (CNS) and perceived feasibility in clinical practice.

**Methods:**

Family caregivers of primary brain tumor patients were recruited from outpatient clinics and asked to complete an adapted version of the CNS consisting of 33 common issues caregivers report (item scale 0–10), and the wish for support (yes/no). Participants ranked acceptability and feasibility (item scale 0–7; higher scores being positive) of the adapted CNS. Descriptive and non-parametric correlational analyses were applied.

**Results:**

Caregivers (*N* = 71) reported 1–33 unmet caregiving needs (*M* = 17.20, sd = 7.98) but did not always wish for support (range 0–28, *M* = 5.82, sd = 6.96). A weak correlation was found between total number of unmet needs and wish for support (*r* = 0.296, *P* = .014). Most distressing items were patients’ changes in memory/concentration (*M* = 5.75, sd = 3.29), patients’ fatigue (*M* = 5.58, sd = 3.43), and signs of disease progression (*M* = 5.23, sd = 3.15).Caregivers most often wished support with recognizing disease progression (*N* = 24), and least often with managing spiritual issues (*N* = 0). Caregivers evaluated acceptability and feasibility of the CNS tool positively (mean scores ranged 4.2–6.2).

**Conclusions:**

Family caregivers experience distress resulting from many neuro-oncology specific needs, but this is not directly related to wish for support. Family caregiver needs screening could be useful to tailor support to suit their preferences in clinical practice.

Owing to the serious nature of primary brain tumors and treatment side-effects, these tumors have a profound impact on the patient and their loved ones.^1,2^ Patients may experience a multitude of symptoms and side-effects that cause them physical limitations, neurocognitive issues, and changes in behavior or personality.^3–7^ Due to the high disease burden, brain tumor patients often need practical and emotional support. This is usually provided by relatives or loved ones, becoming “family caregivers”. Becoming a caregiver can be a rewarding experience.^8^ However, caregiver burden is significant, particularly among those caring for brain tumor patients^9^ with many caregivers reporting feeling overwhelmed and underprepared for this role.^6^ The reported burden among caregivers includes fatigue often from lack of adequate sleep, financial distress, social isolation and physical and emotional health problems including depression.^10–13^

Caregiver burden can lead to unmet needs or distress,^6^ which can fluctuate over time throughout the patient’s disease trajectory. Factors such as tumor location, disease progression, treatment response and the competing demands of daily life impact the current needs of both the patient and their family caregiver.^14^ The emergent nature of family caregiver needs also lends itself to the issue of reporting, as their needs do not remain constant it becomes difficult to track which needs are relevant in a specific time frame.^6^ What may have been considered a most significant unmet need at one time can change to least concern as the disease progresses and both patient and family caregiver learn to adapt.^15^ However, a few constant requirements emerge: caregivers express the need for more information and support about providing daily care, disease prognostic information, accessing financial support, the ability to manage patient behavior and handling changes in personality or behavior.^16,17^ In addition to the consistent requirements and needs that present themselves in neuro-oncology caregivers, there is also the reported long-term impact and burden that effects informal caregivers, which includes ongoing higher levels of anxiety and depression.^18^

Effectively meeting caregiver needs is not only hindered by a lack of evidence-based specific neuro-oncology interventions,^19–21^ but also by a lack of available support resources in clinical practice.^20^ Yet, without a clearer notion of the fluctuating needs of neuro-oncology family caregivers, developing and testing support options is complicated. A first step to breaking this cycle could be through routine monitoring of caregiver needs, in a similar way as has been done successfully in patients with tumors inside and outside the central nervous system.^20,22,23^ The recently developed and validated Caregiver Needs Screen (CNS) tool has been used to assess the level of distress caregivers experience as a result of a range of needs specific to neuro-oncology.^20^ The CNS may be a potential tool for use in clinical practice. Where available locally, referral to support options could follow.

Unmet needs are defined as the discrepancy between services or support necessary to deal with specific caregiving needs, and the lack of availability or accessibility thereof.^24^ In caregivers who experience unmet needs and high caregiver burden, the preparedness for accepting support (if available) can depend upon whether they have the capacity for engaging with support. Research into unmet needs of caregivers for other diseases such as multiple chronic conditions has shown that caregivers are often resistant to accepting support services for a range of reasons including, the desire to remain independent, fear, and avoiding disruptions in the home.^25^ Expectations regarding timing and accessibility of support resources, and whether these are tailored to the neuro-oncology situation, can also impact neuro-oncology caregivers. There is, however, very little research available that distinguishes between unmet needs and the actual wish for support. Therefore, we aimed to (1) identify the presence and magnitude of unmet needs in family caregivers; (2) examine the associations between unmet needs and wish for support; and (3) gauge acceptability and feasibility of the screen’s use with caregivers. If considered acceptable, routine monitoring of caregiver needs could inform improvements in resources to support family caregivers and the development of new interventions.

## Methods

The present investigation is a mixed-methods, single-center cross-sectional study with a sample of adult caregivers of primary brain tumor patients, to aid understanding of the complexities of family caregiver needs screening in clinical practice.

### Participants

We invited patient-caregiver dyads to participate in this cross-sectional study if (1) they had been diagnosed with a primary brain tumor (any type); (2) both patient and caregiver were over 16 years old; (3) the caregiver was the primary provider of emotional and/or physical support to the patient. Dyads were excluded from participation if (1) they did not speak/read English sufficiently to complete study outcomes; (2) either they or the patient did not sign informed consent. All patients had received their diagnosis and were under treatment or in follow-up after treatment at the Edinburgh Centre for Neuro-Oncology. The study was approved by the NHS Lothian Research Ethics Committee (15/SS/0136).

### Procedure

Study information was posted to potential participants ahead of a scheduled outpatient clinic visit. A reply card was included where dyads could indicate if they agreed to be contacted by a member of the study team, or if applicable, why they would rather not participate. Reminder letters were sent to those dyads who did not respond to the study information after 4 weeks. The study was discussed with potential participants during their clinic visit before written consent from both patient and caregiver was obtained. For patients, participation entailed permission to extract information from their medical records. Caregivers were asked to complete the outcome measures listed below either in the clinic or at home (to be returned by post).

### Outcome Measures

Sociodemographic data were collected through a study-specific questionnaire completed by caregivers. Clinical data were extracted from patients’ medical records.

Caregiver needs were assessed with the *Caregiver Needs Screen* (CNS) (provided as Supplementary material).^20^ The CNS assesses distress resulting from 30 common needs/issues on a 0 (not at all distressed) to 10 (as distressed as you can imagine) scale. The CNS was first piloted to evaluate preliminary caregiver acceptability in a clinical setting, and based on feedback from the pilot study we made some adaptations to the CNS. Adaptations included clarification of the written instructions to emphasize that the questions refer to caregivers’ current situation, minor rephrasing of items, and addition of three items (“changes in memory and concentration”, “dealing with an uncertain future”, and “communicating about the patients” condition’, data only available from a subset of respondents) which resulted in the total reported number of needs being between 0 and 33. Tick boxes were added to each item to assess whether caregivers would like to receive information or advice on supportive care options for that need. Space for remarks regarding any additional concerns was provided. The items on the CNS represent six underlying constructs, (1) neurologic symptoms; (2) oncologic symptoms; (3) personal communication (including talking to friends and family); (4) communicating with healthcare providers; (5) resources; and (6) caregiver health. A total number of distressing needs (ie, unmet needs) and a total number of needs with a wish for support can be assessed.

A brief study-specific evaluation questionnaire was administered to assess the acceptability and feasibility of the CNS in clinical practice. The evaluation questionnaire consisted of a 7-point Likert scale covering three main areas (1) ease of use; (2) usefulness; (3) satisfaction; along with these topic areas the questionnaire asked for participants overall impression of the CNS and for their opinions how they receive advice and support and for any suggestions of improvement on the CNS.

### Statistical Analysis

All analyses were done using SPSS software version 24. Descriptive statistics were used to describe the sample (sociodemographic and clinical characteristics), as well as the presence and magnitude of needs and desire for information/advice on supportive care options (aim 1). To examine associations between magnitude of needs and wish for information/advice, point-bi-serial correlations were run for each item as well as for a total needs score and total wish for support score (aim 2). Correlation coefficients were considered to be very weak (0–0.19), weak (0.2–0.39), moderate (0.4–0.59) strong (0.6–0.79) or very strong (0.8–1.0).^26^ Non-parametric tests (Mann–Whitney *U* or Kruskall Wallis as appropriate) were done to explore associations between the total sum of unmet needs and the total sum of wish for support, and sociodemographic/clinical characteristics [caregiver age (above or below median); sex (male or female); relationship to patient (spouse or other); tumor grade (low- or high-grade); disease stage (under treatment; disease progression/in palliative care; stable disease/in follow-up)]. *P*-values of ≤ .05 were considered statistically significant as standard in scientific literature.^27,28^ Due to the exploratory nature of the study, we did not correct for multiple testing. Finally, descriptive statistics were used to analyze responses to the evaluation questionnaire, and an inductive thematic analysis based on Braun and Clarke’s^29^ 6 step process to examine and generate key thematic findings was used to analyze the free text responses (aim 3). Missing data were not imputed from the subset of results available.

## Results

### Participants

A total of 179 patient-caregiver dyads were invited to participate between January and August 2016. In total, 89 (49.7%) consented and of those, 71 dyads (79.8%) completed study procedures. Fifty-three dyads (29.6%) did not reply and 37 (20.7%) declined participation, with reasons including: bad timing (eg, too much already going on, disease progression; *N* = 7), not interested (*N* = 5), do not wish to be confronted with disease, their issues, or research studies (*N* = 5), do not feel study is relevant to them (*N* = 4).

Participant characteristics are displayed in Tables 1 and 2. No statistically significant differences between participants and non-participants were found in terms of patients’ age and sex. The average age of participating family caregivers was 55.4 years (SD =13.2), and the majority of the caregivers were women (*N* = 43; 60.61%). Most caregivers (*N* = 48; 68.6%) were the spouse of the patient. The most commonly reported patient diagnoses included astrocytoma (28.2%), glioblastoma (19.7%), and meningioma (19.7%). On average, it took caregivers 12.2 min to complete the CNS (SD = 6.7, range 2–40 min).

**Table 1.** Caregiver and patient characteristics

	Participant (*N* = 71)
Caregiver age *M* (sd), range	55.4 (13.2), 19–84
Caregiver sex *N* [%]	
Male	28 [39.4%]
Female	43 [60.6%]
Caregiver educational level *N* [%]	
Primary school	1 [1.4%]
Lower secondary school	18 [26.1%]
Upper secondary school	11 [15.9%]
University or college below a degree	17 [24.6%]
University or college degree	22 [31.9%]
Marital status *N* [%]	
Single, never married	4 [5.6%]
Married or living together	62 [87.3%]
Separated	2 [2.8%]
Divorced	2 [2.8%]
Widowed	1 [1.4%]
Relationship with patient *N* [%]	
Spouse	48 [68.6%]
Sibling	2 [2.9%]
Parent	13 [18.6%]
Child	3 [4.3%]
Other (partner or co-habitee)	4 [5.7%]
Patient age *M* (sd), range	51.7 (15.1), 19–81
Patient sex *N* [%]	
Male	38 [53.5%]
Female	33 [46.5%]
Patient tumor type *N* [%]	
Ependymoma	2 [2.8%]
Oligodendroglioma	12 [16.9%]
Astrocytoma	20 [28.2%]
Glioblastoma	14 [19.7%]
Meningioma	14 [19.7%]
Medulloblastoma	2 [2.8%]
Other*	7 [9.9%]
Patient tumor grade *N* [%]	
WHO grade I	13 [20.6%]
WHO grade II	18 [28.6%]
WHO grade III	17 [27.0%]
WHO grade IV	15 [23.8%]
Patient treatment *N* [%]	
Biopsy	12 [16.9%]
Resection	58 [81.7%]
Chemotherapy	26 [36.6%]
Radiotherapy	56 [78.9%]
Disease phase *N* [%]	
Shortly after diagnosis	1 [1.4%]
Under treatment	9 [12.7%]
Disease progression	7 [9.9%]
Stable disease/follow-up	52 [73.2%]
Palliative care	1 [1.4%]
Rehabilitation	1 [1.4%]

*Primary CNS lymphoma; craniopharyngioma; pineal parenchymal tumor of intermediate differentiation; haemangiopericytoma; choroid plexus carcinoma; brainstem glioma; likely glioma without confirmed histopathology; optic nerve glioma; chondrosarcoma; presumed germinoma or colloid glioma; pineal germinoma; desmoplastic infantile ganglioma; hypothalamic glioma; astroblastoma.

**Table 2.** Diagnosis and malignancy grade breakdown

	Malignancy grade				Total
	Grade 1	Grade 2	Grade 3	Grade 4	
Diagnosis					
Oligodendroglioma	0	8	4	0	12
Astrocytoma	5	6	9	0	20
Glioblastoma	0	0	0	14	14
Ependymoma	0	2	0	0	2
Meningioma	6	1	3	0	10
Medulloblastoma	0	0	0	1	1
Other	2	1	1	0	4
Total	13	18	17	15	63

*Malignancy grade data missing for eight cases.

### Presence and Extent of Unmet Needs

Table 3 provides an overview of caregivers’ unmet needs and their wish for support. The highest levels of unmet needs generally fell within the neurological domain. The highest reported concern was changes in patient’s “memory or concentration” (*M* = 5.75, SD = 3.29; subset of *N* = 29 responses). This was followed closely by patient’s “Fatigue or tiredness” (*M* = 5.58, SD = 3.43), “Recognizing signs of disease progression” (*M* = 5.23, SD = 3.15), “Changes in thinking or behavior” (*M* = 5.04, SD = 3.50), “Patient distress or sadness” (*M* = 4.68, SD = 3.41), “Dealing with uncertain future” (*M* = 4.46, SD = 3.51) and “Negative changes in caregivers” own emotional health’ (*M* = 4.44, SD = 3.49).

**Table 3.** Overview of caregiver needs and wish for information/advice and corresponding correlation coefficients

	*N*	*M*, range	Requested information or advice (%)	Correlation coefficient, *P*-value
Neurologic symptoms				
Changes in relationship with the patient	70	2.69 (0–10)	7.14	*r*_pb_ = .394 *P* = .001
Recognizing signs of disease progression	69	5.23 (0–10)	34.78	*r*_pb_ = .299 *P* = .007
Changes in thinking or behavior	68	5.04 (0–10)	19.11	*r*_pb_ = .281 *P* = .017
Distress or sadness	69	4.68 (0–10)	20.28	*r*_pb_ = .277 *P* = .015
Difficulty speaking	69	2.49 (0–10)	11.59	*r*_pb_ = .395 *P* = .001
Weakness	68	3.09 (0–10)	8.82	*r*_pb_ = .297 *P* = .012
Change in vision	69	2.74 (0–10)	17.39	*r*_pb_ = .297 *P* = .012
Numbness	68	1.93 (0–10)	13.23	*r*_pb_ = .488 *P* = .000
Pain	70	3.40 (0–10)	14.28	*r*_pb_ = .494 *P* = .000
Seizures	69	3.57 (0–10)	18.84	*r*_pb_ = .542 *P* = .000
Oncologic symptoms				
Nausea/vomiting	68	2.21 (0–10)	5.88	*r*_pb_ =.230 *P* = .060
Change in appearance	68	2.29 (0–10)	8.82	*r*_pb_ = .361 *P* = .003
Shortness of breath	69	1.49 (0–10)	8.69	*r*_pb_ = .310 *P* = .015
Disturbed sleep	69	3.90 (0–10)	21.73	*r*_pb_ = .493 *P* = .000
Fatigue or tiredness	69	5.58 (0–10)	21.73	*r*_pb_ = .421 *P* = .000
Lack of appetite	69	2.59 (0–10)	20.28	*r*_pb_ = .580 *P* = .000
Changes in bowel pattern	67	2.0 (0–10)	13.43	*r*_pb_ = .538 *P* = .000
Personal communication				
Communicating with family and friends	70	2.67 (0–10)	4.28	*r*_pb_ = .221 *P* = .060
Talking to (grand)children	67	1.76 (0–10)	2.98	*r*_pb_ = .193 *P* = .125
Communicating with health care providers				
Treatment options	70	4.21 (0–10)	18.57	*r*_pb_ = .470 *P* = .000
Managing medications and side-effects	70	3.99 (0–10)	25.71	*r*_pb_ = .412 *P* = .000
Resources				
Financial issues	70	3.20 (0–10)	8.57	*r*_pb_ = .354 *P* = .002
Employment benefits and legal issues	70	2.77 (0–10)	12.85	*r*_pb_ = .511 *P* = .000
Obtaining services for your loved one	70	3.14 (0–10)	14.28	*r*_pb_ = .485 *P* = .000
Arranging or managing transportation	70	2.49 (0–10)	8.57	*r*_pb_ = .352 *P* = .003
Obtaining child care	67	0.64 (0–10)	5.97	*r*_pb_ = .433 *P* = .001
Managing spiritual issues	68	0.32 (0–8)	0	*N/A*
Managing nutrition	69	2.69 (0–10)	18.84	*r*_pb_ = .529 *P* = .000
Caregiver health				
Changes in your physical health	67	3.52 (0–10)	10.44	*r*_pb_ = .330 *P* = .005
Changes in your emotional health	68	4.44 (0–10)	17.64	*r*_pb_ = .316 *P* = .006
Additional items				
Changes in memory or concentration	29	5.75 (0–10)	27.6	*r*_pb_ = .185 *P* = .26
Dealing with uncertain future	29	4.46 (0–10)	17.2	*r*_pb_ = .436 *P* = .010
Communicating about patients’ condition	29	4.25 (0–10)	17.2	*r*_pb_ = .565 *P* = .001

For each CNS item, family caregivers indicated whether they would like to receive information or advice on support options. This was most frequently indicated for “Recognizing signs of disease progression” (*N* = 24, 34.78%), “Managing medications and side-effects” (*N* = 18, 25.71%), “Fatigue or tiredness” (*N* = 15, 21.73%), “Disturbed sleep” (*N* = 15, 21.73%), “Distress or sadness” (*N* = 14, 20.28%), and “Lack of appetite” (*N* = 14, 20.28%). The mean total unmet needs score of the caregiver sample was *M* = 17.2 out of 33 (SD = 7.9), and the mean total score for the wish to receive information on these unmet needs was *M* = 5.82 out of 33 (SD = 6.96). The sum of unmet needs and the items for which caregivers indicated a wish for support were not associated with caregiver age, sex, relationship to patient, tumor grade, or disease stage  (all *P* > .05).

### Associations Between Unmet Needs and Wish for Information/Advice

The final column of Table 3 shows results of point bi-serial correlations between each need and wish for information/advice. In general, we found weak to moderate correlations between the CNS items and the wish for receiving information on support: ranging from *r*_pb_ = .185 for “changes in memory or concentration” to *r*_pb_ = .58 for “lack of appetite”. The strongest associations between unmet needs and wish for support were of moderate strength and focused within the symptom-based constructs (oncological and neurological), *r*_pb_ = .58 for “lack of appetite”, *r*_pb_ = .53 for “change in bowel pattern” and *r*_pb_ = .52 “for managing nutrition”, *r*_pb_ = .57 for “change in vision” and *r*_pb_ = .54 for seizures. This indicates that symptom-based unmet needs among family caregivers garner a relatively strong desire for information or support in managing these concerns. An overall examination of the association between the total level of unmet needs and the wish for support revealed a weak positive correlational relationship (*r*_pb_ = .29), indicating that there is a weak link between unmet needs and a caregiver’s wish for support with these issues.

### Caregivers’ Perceived Acceptability and Feasibility of the Caregiver Needs Screen (CNS) in Clinical Practice

Family caregivers were asked to evaluate the CNS by rating the tool from 1 to 7 based on a number of categories including: “ease of use”, “usefulness” and “satisfaction” within a clinical environment. They were also asked to consider their overall impression of the tool and to indicate how they would like to receive information on potential support options. Responses to the caregiver evaluation form are presented in Table 4. Overall, family caregivers evaluated the CNS tool positively with mean scores for all items on the evaluation form ranging from 4.19 to 6.21 out of 7. The highest scores were for items asking whether caregivers felt there were no inconsistencies (*M* = 6.21, SD = 1.07), whether the CNS does not have more questions than necessary (*M* = 6.10, SD = 1.20), ease of use (*M* = 5.98, SD = 1.25), and helpfulness in an outpatient setting (*M* = 5.62, SD = 1.38). These responses may indicate that caregivers find the tool acceptable for use in clinical practice. The preferred delivery method for information/advice on support options was mixed. Many preferred a combination of the delivery methods listed (*N* = 18, 30.0%), followed by email (*N* = 17, 28.3%), on paper (*N* = 12, 20.0%), in person (*N* = 6, 10.0%), and by telephone (*N* = 2, 3.3%). Often, caregivers indicated to want to receive this information from a doctor or nurse (*N* = 32, 53.3%), with only some listing researchers (*N* = 6, 10.0%) or volunteers and past patients (*N* = 2, 3.3%).

**Table 4.** Overview of perceived acceptability

Perceived acceptability of the CNS	*N*	*M*, range, SD
It is easy to use the questionnaire	66	5.98 (1–7) 1.25
The questionnaire’s format is user friendly	66	5.81 (1–7) 1.42
It does not have more questions than necessary	65	6.10 (2–7) 1.20
Completing the questionnaire is effortless	65	5.66 (1–7) 1.50
I can use it without written instructions	66	5.74 (1–7) 1.73
I don’t notice any inconsistencies as I use it	66	6.21 (2–7) 1.07
I can recover from mistakes (in answers) quickly and easily	56	5.91 (2–7) 1.25
The questionnaire helps me express my needs as a caregiver	65	5.53 (2–7) 1.29
I believe it could help me find my way to supportive care if I need it	65	5.41 (1–7) 1.61
I find this questionnaire useful	65	5.41 (2–7) 1.49
It gives me more insight into my needs as a caregiver	64	4.81 (1–7) 1.76
I can see this being helpful in the hospital setting	64	5.62 (1–7) 1.38
I am satisfied with the questionnaire	65	5.75 (2–7) 1.31
I would recommend it to other caregivers	61	5.62 (1–7) 1.58
The format of the questionnaire is to my liking	64	5.62 (1–7) 1.44
I feel I need to have access to this on a regular basis	62	4.19 (1–7) 1.99
Overall impression of the questionnaire	65	5.60 (1–7) 1.29

### Open-ended Questions: Caregiver Concerns

Caregivers were asked to remark on their experiences, providing any context or listing any other concerns they may have. We collated the remarks into key themes which were then categorized by frequency (see Table 5). Caregivers indicated that their key concerns were focused on “future concerns” (*N* = 7) and the “communication of a patients diagnosis, treatment or symptoms” (*N* = 6). These remarks included concerns around the patient’s future prospects.

**Table 5.** Themes emerging from free text responses

Themes	Number	Percent
Theme 1: Future concerns	7	31.0
Theme 2: Financial implications	3	13.0
Theme 3: Deterioration of caregiver health	4	17.0
Theme 4: Communication of diagnosis/treatment/symptoms	6	26.0
Other comments*	3	13.0

*Other comments include remarks about no current issues or the general dislike for filling out forms.

Uncertainty about patient’s future; now he is unemployed how he spends his time; no friends/social life.

In particular, the patients’ future employment and social life were of significant concern; a future without the presence of the family caregiver was a key feature in the caregiver remarks.

My biggest concern is that should something happen to me, what would happen to [patient name]. It upsets me that [patient name] has a low tolerance in the company of family and grandchildren, he has a very low tolerance to a lot of noise and chatter.

Other key concerns centered on the communication of the patient’s situation and their current symptoms to health care professionals.

We were never ever been given support and just left to fend for ourselves. Patient also has other undiagnosed symptoms and we are not getting anywhere with results which leave us feeling very down and alone.

Family caregivers’ unmet needs or concerns remain present throughout the progression of a patient’s disease. The remarks expressed by caregivers indicate that they face mutual unmet needs and concerns around the patient’s future and their disease progression.

## Discussion

This study explored neuro-oncology caregiver unmet needs, as well as associations between these needs and the wish for information, advice, and support. Most caregivers reported unmet needs related to patients’ neurological and oncologic symptoms, as well as changes in their own emotional health. This is in line with existing literature^17,30–33^ which highlights similar areas of concern and unmet support needs in neuro-oncology caregivers: obtaining information and practical support; dealing with uncertainty and worries; having time for yourself; understanding the patient’s illness and managing their symptoms.^1,32,34^ Interestingly these unmet needs crossover into other neurological diseases or conditions such as acquired brain injury and dementia,^35^ who all report similar levels of burden to cancer caregivers. Despite the similarities of neurological caregivers facing high levels of burden, it is important to highlight that each disease group has its own unique set of challenges and these must be addressed from a specialist level providing tailored support.^35^

Other studies highlight that there is a lack of guidance in accessing relevant information^31^ as well as a lack of timely access to good quality support services.^30^ Yet, our report underscores the importance of not just investigating areas of unmet need but also, caregivers’ wish or desire for engaging with support—as the two concepts do not always overlap.

The issues with which caregivers most frequently wanted support corresponded generally to those areas of greatest distress, but correlations were of weak to moderate strength, indicating this is not a straightforward linear relationship. However, it does highlight with which areas caregivers want to receive help rather than strategies such as accessing existing services, coping on their own, or relying on support from friends and family. Lageman et al^36^ similarly found that in a sample of 32 neuro-oncology caregivers, support needs were low to moderate on average, but emphasized that looking at averages masks the fact that there are subgroups with very high needs who might benefit more from support. A survey completed by 70 neuro-oncology caregivers showed that those with higher-than-average supportive care needs and greater emotional distress expressed greater interest in support services.^37^ In our study, only a weak correlation was found between the total level of unmet needs and a wish for information/advice or support. In part, this may have been influenced by our participant selection criteria and recruitment method, which are purposefully broad and inclusive to closely mirror the typical neuro-oncology clinic, and limit (self-)selection bias. In future efforts we suggest focusing on neuro-oncology caregiver subgroups with very high support needs.,

As we intended to explore whether the CNS could be feasible for use as a tool to assess unmet needs and wish for support in clinical practice, we invited participants to evaluate the tool based on a number of categories including; its “ease of use”, “usefulness”, and “user satisfaction”. Participants evaluated the screening tool favorably, indicating that caregivers may find the use of the CNS acceptable and feasible within a clinical setting. Completion times averaged at 12 min. This is within the generally accepted time frame of 20 min for survey completion.^38^ The CNS could provide a pragmatic, quick, and easy-to-use neuro-oncology specific alternative to other tools, such as the Carer Support Needs Assessment Tool (CSNAT) which is caregiver-led, and facilitated by a healthcare professional. The CSNAT has been trialed in palliative cancer settings,^39,40^ with subgroup analyses reported for neuro-oncology caregivers.^34^ Similarly, the CNS has been used as part of a nurse-led, needs-based neuro-oncology specific intervention (SmartCare), which was found effective in decreasing caregiving-specific distress.^41^ Yet, our study highlights that despite such efforts, unmet needs persist in caregivers accompanying patients to routine neuro-oncology clinic visits.

Even in cancer patient populations, implementation of screening or support referrals is not without issues.^42–44^ Dekker et al^45^ suggest that a fundamental change in the management of patient’s emotional health is required to counter the mismatch between patient needs and provision of support. The same is likely true for successful implementation of needs screening and support for caregivers. Still, the routine use of screening tools to effectively manage patient and family caregiver quality of life has been recommended as part of a larger program of integrated healthcare that includes, regular and routine screening tools alongside appointing a case manager to map out brain tumor trajectories, with the aim of reducing both caregiver specific and patient distress.^46^ We envisage that the CNS tool can play its part by helping identify the areas individual in which caregivers wish for support in clinical practice. Treatment teams or general practitioners may then be able to provide tailored support or refer to caregiver specific resources, such as signpost caregivers to community based support groups or include them in discussions about providing care from diagnosis and help them develop coping strategies.^47^

This study has its limitations. It was a single-center, cross-sectional study and therefore may not be representative of other neuro-oncology clinics, or reflect the changing needs of caregivers over time. We aimed to sample caregivers of primary brain tumor patients (any type) consecutively to closely mimic the population seen in neuro-oncology follow-up clinics, and maximize generalizability of findings as far as possible. However, we acknowledge that disease and caregiver burden varies within patient subgroups, and may vary between younger and older caregivers. In addition, the CNS does not explicitly cover caregiver needs related to grief and mortality. We observed limited consent and completion rates (53.0% and 42.3% respectively). Although participants and non-participants did not statistically differ in age or sex, we acknowledge that research participation is influenced by other socioeconomic, racial, and cultural factors which create a body of “hidden voices” in clinical research, therefore our findings may not be representative of these factors.^48,49^ However, similar studies showed comparable participation rates ranging between 30 and 50%.^37,40^ Finally, the pragmatic adaptations made to the preliminary version of the CNS should be highlighted as a limitation, as the original CNS was validated after the data for the present study had been collected.^18^ In future efforts we recommend using the validated 30-item CNS with the added tick-box to indicate a wish for support and an open-ended text box to list any additional issues (provided as Supplementary material).

In conclusion, this study showed that in consecutively sampled caregivers in a typical neuro-oncology clinic, unmet needs persist despite first studies emerging on this topic well over a decade ago. Correlations between unmet needs and the wish for support are weak to moderate, which highlights the importance of assessing *both* aspects in routine clinical practice so that caregivers may be signposted to available existing resources. The CNS tool is a neuro-oncology specific and pragmatic tool which could be used for precisely this purpose.

## Supplementary Material

npac099_suppl_Supplementary_File
