# Supplementary material for: Unmet needs and wish for support of family caregivers of primary brain tumor patients
Source: Neurooncol Pract. 2023 Jan 4;10(3):271–80. doi: 10.1093/nop/npac099 (PMC10180375; doi:10.1093/nop/npac099)
Supplement: npac099_suppl_Supplementary_File [file npac099_suppl_supplementary_file.pdf]

Neuro-oncology Caregiver Needs Questionnaire

Instructions:

Listed below are a number of issues that caregivers may face as they help care for a loved one with a brain tumour. Thinking about the issues you faced during the **past 2 weeks**, please select the number that best describes how distressed you have been about that issue where **0 = not at all distressed and 10 = as distressed as you can imagine.** If you did not experience that issue, please select 0. Then indicate **if** you wish to receive information about supportive care options to help you manage your stress.

In the past 2 weeks, how distressed have you been about your loved one's…

Issues relating to patients' treatment/s	How much stress do you experience?	Receive information on supportive care?
1. Sleep problems	No distress 0 1 2 3 4 5 6 7 8 9 10 Most distress	Yes, I'd like to receive information ☐
2. Fatigue	No distress 0 1 2 3 4 5 6 7 8 9 10 Most distress	Yes, I'd like to receive information ☐
3. Emotions	No distress 0 1 2 3 4 5 6 7 8 9 10 Most distress	Yes, I'd like to receive information ☐
4. Changes in thinking, behaviour, personality	No distress 0 1 2 3 4 5 6 7 8 9 10 Most distress	Yes, I'd like to receive information ☐
5. Difficulty speaking	No distress 0 1 2 3 4 5 6 7 8 9 10 Most distress	Yes, I'd like to receive information ☐
6. Muscle weakness	No distress 0 1 2 3 4 5 6 7 8 9 10 Most distress	Yes, I'd like to receive information ☐
7. Headaches or other types of pain	No distress 0 1 2 3 4 5 6 7 8 9 10 Most distress	Yes, I'd like to receive information ☐
8. Changes in sensation: loss of feeling or numbness and tingling	No distress 0 1 2 3 4 5 6 7 8 9 10 Most distress	Yes, I'd like to receive information ☐
9. Seizures	No distress 0 1 2 3 4 5 6 7 8 9 10 Most distress	Yes, I'd like to receive information ☐
10. Changes in vision	No distress 0 1 2 3 4 5 6 7 8 9 10 Most distress	Yes, I'd like to receive information ☐
11. Safety Issues	No distress 0 1 2 3 4 5 6 7 8 9 10 Most distress	Yes, I'd like to receive information ☐
12. Changes in appetite	No distress 0 1 2 3 4 5 6 7 8 9 10 Most distress	Yes, I'd like to receive information ☐
13. Bowel problems: constipation, diarrhoea, and incontinence/toileting issues	No distress 0 1 2 3 4 5 6 7 8 9 10 Most distress	Yes, I'd like to receive information ☐
14. Nausea and vomiting	No distress 0 1 2 3 4 5 6 7 8 9 10 Most distress	Yes, I'd like to receive information ☐
15. Blood clots or abnormal bleeding	No distress 0 1 2 3 4 5 6 7 8 9 10 Most distress	Yes, I'd like to receive information ☐
16. Changes in appearance	No distress 0 1 2 3 4 5 6 7 8 9 10 Most distress	Yes, I'd like to receive information ☐
17. Shortness of breath	No distress 0 1 2 3 4 5 6 7 8 9 10 Most distress	Yes, I'd like to receive information ☐
18. Changes in your loved one's disease status	No distress 0 1 2 3 4 5 6 7 8 9 10 Most distress	Yes, I'd like to receive information ☐

Neuro-oncology Caregiver Needs Questionnaire

Instructions: *see previous page*

In the past 2 weeks, how distressed have you been about….

Obtaining information and service issues	How much stress do you experience?	Receive information on supportive care?
1. Managing your loved ones medications and side effects	No distress 0 1 2 3 4 5 6 7 8 9 10 Most distress	Yes, I'd like to receive information ☐
2. Your loved ones treatment options	No distress 0 1 2 3 4 5 6 7 8 9 10 Most distress	Yes, I'd like to receive information ☐
3. Financial, legal or advanced planning issues	No distress 0 1 2 3 4 5 6 7 8 9 10 Most distress	Yes, I'd like to receive information ☐
4. Employment benefits, work-related concerns, or insurance issues	No distress 0 1 2 3 4 5 6 7 8 9 10 Most distress	Yes, I'd like to receive information ☐
5. Finding and obtaining community services and resources	No distress 0 1 2 3 4 5 6 7 8 9 10 Most distress	Yes, I'd like to receive information ☐

Communication Issues	How much stress do you experience?	Receive information on supportive care?
1. Communicating with health care providers	No distress 0 1 2 3 4 5 6 7 8 9 10 Most distress	Yes, I'd like to receive information ☐
2. Communicating with family or friends	No distress 0 1 2 3 4 5 6 7 8 9 10 Most distress	Yes, I'd like to receive information ☐
**3.** Talking with children or grandchildren	No distress 0 1 2 3 4 5 6 7 8 9 10 Most distress	Yes, I'd like to receive information ☐

Taking care of your own needs	How much stress do you experience?	Receive information on supportive care?
1. Maintaining your emotional health	No distress 0 1 2 3 4 5 6 7 8 9 10 Most distress	Yes, I'd like to receive information ☐
**2.** Maintaining your physical health	No distress 0 1 2 3 4 5 6 7 8 9 10 Most distress	Yes, I'd like to receive information ☐

General caregiver issues	How much stress do you experience?	Receive information on supportive care?
1. Changes in the relationship with your loved one	No distress 0 1 2 3 4 5 6 7 8 9 10 Most distress	Yes, I'd like to receive information ☐
2. Spiritual issues	No distress 0 1 2 3 4 5 6 7 8 9 10 Most distress	Yes, I'd like to receive information ☐

Room for remarks

If you have any additional concerns that cause distress, please list these below:

……

……

……

Thank you *for completing this questionnaire!*
